# A Conserved Glycine Is Identified to be Essential for Desaturase Activity of IpFAD2s by Analyzing Natural Variants from *Idesia polycarpa*

**DOI:** 10.3390/ijms19123932

**Published:** 2018-12-07

**Authors:** Pan Wu, Lingling Zhang, Tao Feng, Wenying Lu, Huayan Zhao, Jianzhong Li, Shiyou Lü

**Affiliations:** 1 Key Laboratory of Plant Germplasm Enhancement and Specialty Agriculture, Wuhan Botanical Garden, Chinese Academy of Sciences, Wuhan 430074, China; wupanx@126.com (P.W.); zhanglingling@wbgcas.cn (L.Z.); fengtao@wbgcas.cn (T.F.); luwenying16@mails.ucas.ac.cn (W.L.); 2 University of Chinese Academy of Sciences, Beijing 100049, China; 3 Applied Biotechnology Center, Wuhan Institute of Bioengineering, Wuhan 430415, China; huayan_zhao@163.com; 4 Tianjin Garrison hangu farm, Tianjin 300480, China; lvsy1021@gmail.com; 5 Sino-Africa Joint Research Center, Chinese Academy of Sciences, Wuhan 430074, China

**Keywords:** *Idesia polycarpa var*, glycine, FAD2, linoleic acid, oleic acid

## Abstract

High amounts of polyunsaturated fatty acids (PUFAs) in vegetable oil are not desirable for biodiesel or food oil due to their lower oxidative stability. The oil from *Idesia polycarpa* fruit contains 65–80% (mol%) linoleic acid (C18:2). Therefore, development of *Idesia polycarpa* cultivars with low PUFAs is highly desirable for *Idesia polycarpa* oil quality. Fatty acid desaturase 2 (FAD2) is the key enzyme converting oleic acid (C18:1) to C18:2. We isolated four FAD2 homologs from the fruit of *Idesia polycarpa*. Yeast transformed with *IpFAD2-1*, *IpFAD2-2* and *IpFAD2-3* can generate appreciable amounts of hexadecadienoic acid (C16:2) and C18:2, which are not present in wild-type yeast cells, revealing that the proteins encoded by these genes have Δ^12^ desaturase activity. Only trace amounts of C18:2 and little C16:2 were detected in yeast cells transformed with *IpFAD2-4*, suggesting IpFAD2-4 displays low activity. We also analyzed the activity of several FAD2 natural variants of *Idesia polycarpa* in yeast and found that a highly conserved Gly376 substitution caused the markedly reduced products catalyzed by IpFAD2-3. This glycine is also essential for the activity of IpFAD2-1 and IpFAD2-2, but its replacement in other plant FAD2 proteins displays different effects on the desaturase activity, suggesting its distinct roles across plant FAD2s proteins.

## 1. Introduction

Vegetable oils are not only essential resources for nutritional applications, but also for sustainable industrial feedstocks, which are commonly used in paints, lubricants, soaps, biodiesel, etc. [1,2]. The demand for vegetable oils is quickly increasing due to the fast growing population across the world. To meet this demand, many efforts have been made to improve the yields of oil crops or to domesticate wild oilseed plants [3]. *Idesia polycarpa,* a member of *Flacourtiaceae* family, is a local tree species in some Asian countries including Korea, Japan, and China [4]. It is receiving more attention due to the high amount of oil in its fruits, which can potentially be used in the biodiesel industry [4]. In addition, the oil from *Idesia polycarpa* fruit is healthy and edible since it contains 65–80% (mol%) linoleic acid (C18:2). C18:2 is one of the essential polyunsaturated fatty acids in humans and cannot be synthesized by the human body, it can only be obtained from food [5]. However, high amounts of C18:2 in its oil also make it more prone to rancidity and thus decreases its flavor [6]. Previous reports showed that oleic acid (C18:1) had higher oxidative stability than C18:2, and thus the edible oils with higher ratios of C18:1/C18:2 are more desirable [7]. Similarly, an ideal biodiesel composition should also contain more monounsaturated fatty acids and less polyunsaturated fatty acids since high percentages of polyunsaturated fatty acids in biodiesel negatively affect its oxidative stability and cause high rates of nitrogen oxide emission [8]. Hence it would be valuable to breed *Idesia polycarpa* cultivars which produce oils with low C18:2 and high C18:1 contents and it would also be helpful to uncover the desaturation mechanism in woody plants.

The ∆^12^ fatty acid desaturase 2 (FAD2) is the key microsomal enzyme that converts C18:1 to C18:2 [9]. Many efforts have been made to modify the plant oil quality via manipulation of expression levels of FAD2, or through screening natural varieties with altered FAD2 activity. For example, the mutations of GmFAD2-1A and GmFAD2-1B greatly increased the levels of C18:1 in soybean seeds [10]. The *Arachis hypogaea AhFAD2* mutant was used as an introgression line for breeding peanut cultivars with high C18:1 and low C18:2 [11]. Numerous safflower breeding lines with high levels of C18:1 (75–84%) were selected from the natural variations in *FAD2* genes [12]. The mutation of a candidate protein, fatty acid desaturase-2 (FAD2-1D) gene from pima cotton produced less linoleic acid [13,14]. Olive oil extracted from the olive fruit has mostly high level of C18:1 (about 75%) and less C18:2 (about 5.5%), which might be attributed to the suppression of *FAD2* genes by si-RNA [15]. Taken together, the activity of FAD2 is crucial for determining the C18:1/C18:2 ratios in seed or fruit storage lipids and FAD2 is an ideal candidate for improving oil quality of oil crops or trees.

Plant FAD2 proteins belong to a large family of ER localized membrane-bound desaturases [16]. The relationship between structure and function of FAD2 proteins has been extensively studied in the past two decades. FAD2 proteins contain three to six predicated transmembrane domains (PTMDs) and three highly conserved histidine-rich motifs, which are key characteristics of all membrane-bound desaturases. In the conserved histidine-rich motifs, the histidines are proved to be crucial for FAD2 desaturase activity [17]. In addition, four relevant amino acid residues within a distance of five residues from the His boxes of AtFAD2 are responsible for the conversion of this monofunctional desaturase (∆^12^ desaturase activity) into a bifunctional desaturase/hydroxylase [18]. Besides these His boxes, McCartney et al. [19] found that the deletion of *Arabidopsis thaliana* AtFAD2 C-terminus containing an ER retrieval motif resulted in loss of ER localization and enzyme activity in yeast cells. In addition, Hoffmann et al. [20] showed that a small membrane-peripheral region close to the active center of a monofunctional Δ^12^ desaturase from *Aspergillus nidulans* determines substrate specificity and regioselectivity. Despite much progress in the relationship between FAD2s structure and function, which has been elucidated in the past two decades, some unidentified factors affecting their enzymatic activity remain to be investigated.

C18:2 was the major component of fatty acids in both seed and pericarp of *Idesia ploycarpa*, which respectively accounted for 83.92% and 62.08% of the total fatty acids in the two organs [5]. Our previous study showed that four *IpFAD*2 genes exist in *Idesia ploycarpa* [5]. To ascertain if the IpFAD2 proteins are capable of desaturating C18:1 into C18:2, we isolated four *IpFAD2* genes from *Idesia ploycarpa*, and identified their activity in yeast cells. We also assessed the activity of natural FAD2 variants and identified a highly conserved glycine at position 376 of IpFAD2-3, which is critical for normal function of IpFAD2s. To determine if the function of this glycine is conserved across plant FAD2 proteins, we also evaluated the impacts of its substitution on the activity of FAD2 proteins from other plants. Our study provides a clue for genetically modifying the oil quality of *Idesia polycarpa*.

## 2. Results

### 2.1. Isolation of FAD2 Orthologs from Idesia ploycarpa

**Figure 1:**
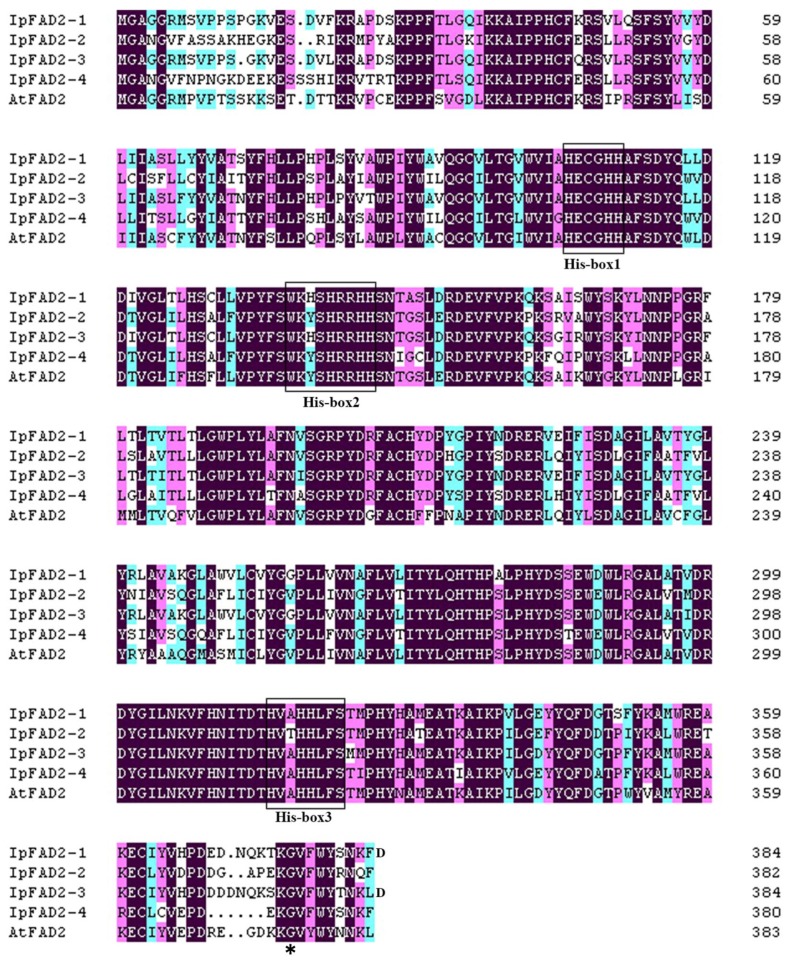
Alignments of predicted amino acid sequences encoded by FAD2 coding sequences from *Idesia polycarpa* and *Arabidopsis thaliana.* The three “histidine-rich motifs” are boxed. The GenBank accession numbers of *IpFAD2-1*, *IpFAD2-2*, *IpFAD2-3, IpFAD2-4, AtFAD2* are: MH394208, MH394209, MH394210, MK105894, and NP_187819.1 accordingly. The shading colors represent the identity level of amino acids. Black, magenta, and cyan indicate 100%, 80%, and 60% identity, respectively. The asterisk indicates the conserved glycine.

Our previous study showed that four *FAD2* orthologs are present in the fruit of *Idesia polycarpa* [5], which were renamed *IpFAD2-1* (c63420_g2), *IpFAD2-2* (c56614_g1), *IpFAD2-3* (c63420_g1), and *IpFAD2-4* (c50543_g1), respectively, in this study. The entire coding sequence (CDS) of *IpFAD2-1*, *IpFAD2-2, IpFAD2-3,* and *IpFAD2-4* were cloned from *Idesia ploycarpa*. The length of the predicted polypeptides encoded by *IpFAD2-1*, *IpFAD2-2, IpFAD2-3,* and *IpFAD2-4* CDS are 385, 382, 385, and 380 amino acids respectively. IpFAD2-1, IpFAD2-2, IpFAD2-3 and IpFAD2-4 shared 76.74%, 70.03%, 76.49% and 67.70% identity with AtFAD2 (Figure 1, Supplementary table S1). Four IpFAD2 proteins contain three conserved histidine-rich motifs, which are commonly present in all membrane-bound fatty acid desaturases (Figure 1) [21,22] and also contain an ER-localized motif in C-terminus [19]. The cDNA sequences of *IpFAD2-1*, *IpFAD2-2*, *IpFAD2-3,* and *IpFAD2-4* were submitted to the NCBI Genbank, their accession numbers were MH394208, MH394209, MH394210, and MK105894, respectively.

**Figure 2:**
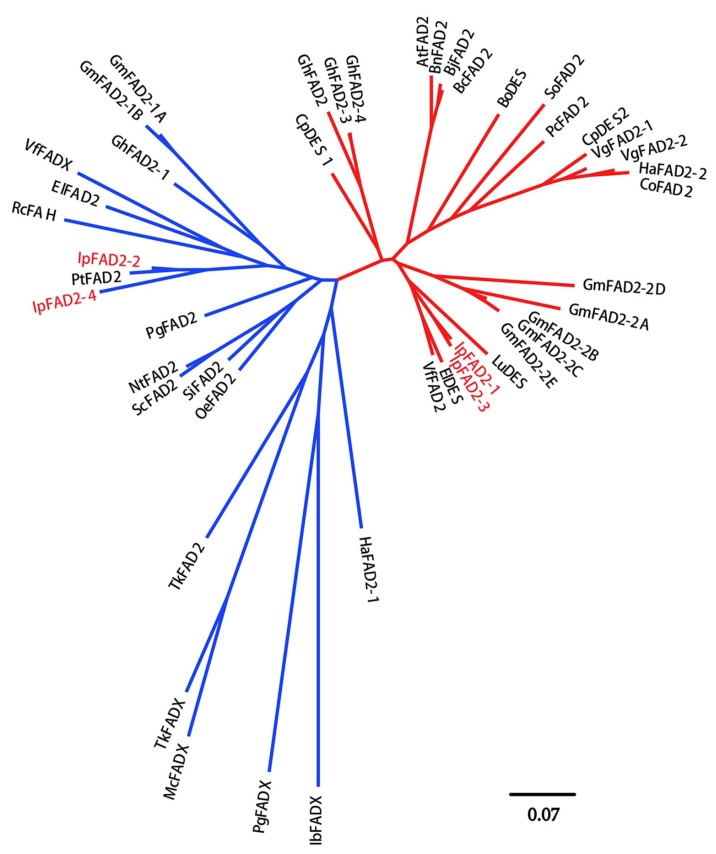
Cladogram of four IpFAD2s and other plant FAD2-related polypeptides. The house-keeping clade and the seed-type clade are labeled in red and blue accordingly. The four FAD2 homologs from *Idesia polycarpa* were designated IpFAD2-1, 2, 3, 4 and were labeled in red. The protein sequences used here were: GmFAD2-1A (Glyma.10G278000.1.p), GmFAD2-1B (Glyma.20G111000.1.p), GmFAD2-2A (Glyma.19G147300.1.p), GmFAD2-2B (Glyma.19G147400.1.p), GmFAD2-2C (Glyma.03G144500.1.p), GmFAD2-2D (Glyma.09G111900.1.p), and GmFAD2-2E (Glyma.15G195200.1.p) from *Glycine max*; CpDES (AAS19533) from *Cucurbita pepo*; IpFAD2-1 (MH394208), IpFAD2-2 (MH394209), IpFAD2-3 (MH394210), and IpFAD2-4 (MK105894) from *Idesia polycarpa*; HaFAD2-1 (AF251842) and HaFAD2-2 (AF251843) from *Helianthus annuus;* IbFADX (AF182520) from *Impatiens balsamina*; PgFADX (AY178446) from *Punica granatum*; McFADX (AF182521) from *Momordica charantia*; TkFADX (AY178444) and TkFAD2 (AY178445) from *Trichosanthes kirilowii;* SiFAD2 (AF192486) from *Sesamum indicum;* ScFAD2 (X92847) from *Solanum commersonii*; RcFAH (EU523112) from *Ricinus communis*; VfFADX (AF525535), and VfFAD2 (AF525534) from *Vernicia fordii*; GhFAD2-1 (X97016), GhFAD2-2 (Y10112), GhFAD2-3 (AF331163), and GhFAD2-4 (AY279315) from *Gossypium hirsutum;* PgFAD2 (AJ437139) from *Punica granatum*; CpDES (AAS19533, and CpDES2 (AAS19533) from *Cucurbita pepo*; AtFAD2 (L26296) from *Arabidopsis thaliana*; BnFAD2 (AF243045) from *Brassica napus*; BjFAD2 (X91139) from *Brassica juncea*; BcFAD2 (AF124360) from *Brassica carinata*; BoDES (AF074324) from *Borago officinalis*; SoFAD2 (AB094415) from *Spinacia oleracea*; PcFAD2 (U86072) from *Petroselinum crispum*; VgFAD2-1 (AF188263) and VgFAD2-2 (AF188264) from *Vernonia galamensis*; CoFAD2 (AF343065) from *Calendula officinalis*; LuDES (ACF49507) from *Linum usitatissimum*.

The phylogenetic relationship of the four IpFAD2s with other reported FAD2s was further elucidated. Similar to the previous report, the selected FAD2 proteins were grouped into two major clades, the house-keeping type and seed-type [16] (Figure 2). House-keeping type FAD2s are constitutively and abundantly expressed, while seed-type FAD2s are specifically or highly expressed in developing seeds [16]. As shown in Figure 2, IpFAD2-1 and IpFAD2-3 belong to the house-keeping group, whereas IpFAD2-2 and IpFAD2-4 fall into the seed-type group.

**Figure 3:**
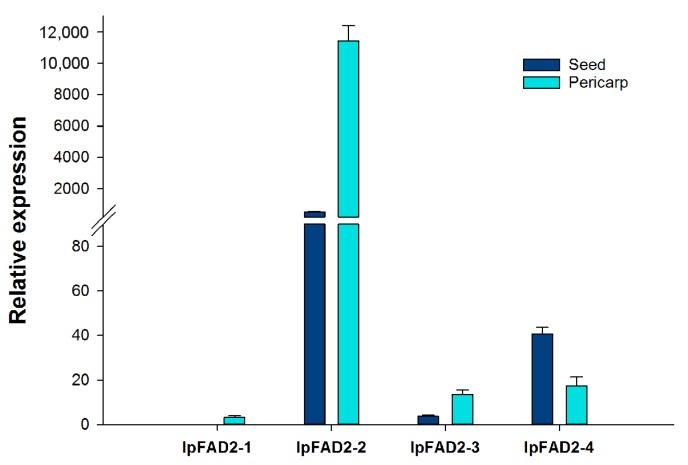
Relative expression levels of IpFAD2 genes in the pericarp and seed of I*desia* polycarpa at 80 days post pollination. Relative expression values were calculated using the 2−ΔΔ*C*t method by using *EF1A* gene as an internal control.

To identify which gene participated in the production of C18:2 content in fruit, we examined the expression patterns of these genes by RT-PCR analysis in seed and pericarp from fruit 80 days after pollination (DAP) (Figure 3). The expression levels of *IpFAD2-2* far exceeded those of the other *IpFAD2* genes, suggesting *IpFAD2-2* might be mainly responsible for producing high C18:2 content in fruit (Figure 3).

### 2.2. Three IpFAD2s Possess Desaturase Activity

**Figure 4:**
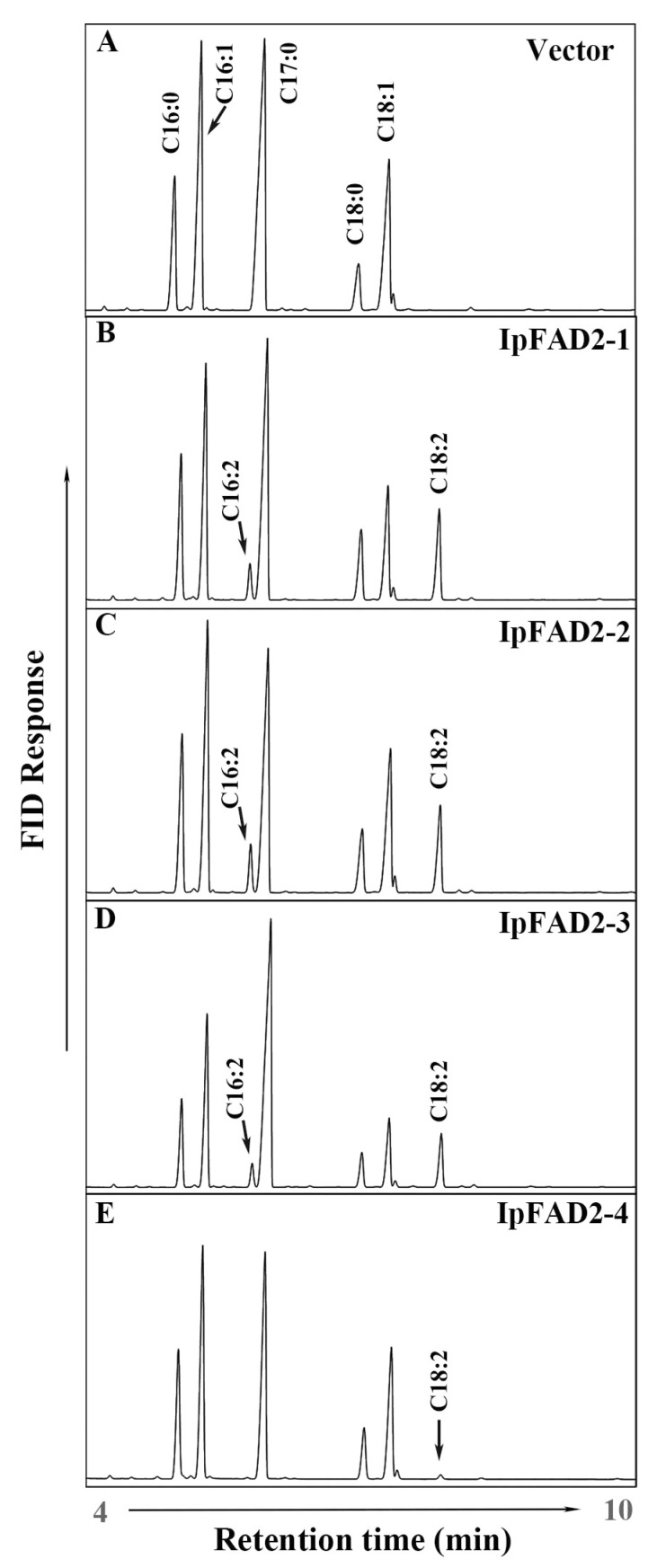
GC analysis of fatty acid methyl esters (FAMEs) isolated from yeast cells expressing IpFAD2s. The FAMEs of total lipid were extracted from yeast transformed with control empty control vector pESC-his (**A**), IpFAD2-1 (**B**), IpFAD2-2 (**C**), IpFAD2-3 (**D**), and IpFAD2-4 (**E**) under induction conditions and analyzed by gas chromatography/flame ionization detector (GC/FID). Major fatty acids peaks are labeled. The newly synthesized fatty acids corresponding to C16:2 Δ9,12 and C18:2 Δ9,12 are indicated by the arrows. Heptadecanoic acid methyl ester (C17:0) is used as the internal standard.

To examine if the proteins encoded by *IpFAD2-1*, *IpFAD2-2*, *IpFAD2-3,* and *IpFAD2-4* are involved in the desaturation process, four genes were transformed into the budding yeast *S. cerevisiae* INVSc1. Then we checked the expression levels of these transgenes by RT-PCR. As shown in Supplementary Figure S1, all transgenes are highly expressed. The fatty acid compositions in the transformed yeast cells were also analyzed by gas chromatography (GC) and the corresponding fatty acids were confirmed by gas chromatography mass spectrometry (GC-MS) (Supplementary Figure S2). The yeast cells harboring *IpFAD2-1, IpFAD2-2* and *IpFAD2-3* produced two novel fatty acids, C16:2 and C18:2, which were not generated in the yeast cells containing blank vector (Figure 4A–D, Supplementary Figure S3), suggesting that the three IpFAD2s have their own catalytic activity. Moreover, the proportion of C18:2 in yeast cells transformed with *IpFAD2-1*, Ip*FAD2-2,* and *IpFAD2-3* is 9.97%, 8.96% and 11.43% of the total fatty acids, much higher than that of C16:2, which only accounts for 2.58%, 4.62%, and 4.45%, respectively. However, only trace amounts of C18:2 (0.66%) and little C16:2 were detected in the yeast cells containing *IpFAD2-4* (Figure 4E). These data indicate that IpFAD2-1, IpFAD2-2, and IpFAD2-3 possess high Δ^12^-fatty acid desaturation activity using both C16:1 and C18:1 as substrates, furthermore C18:1 is a preferable substrate for all three IpFAD2 proteins, while IpFAD2-4 displays low activity (Figure 4A–E).

### 2.3. A Highly Conserved Glycine Residue Identified from the FAD2 Natural Variation is Required for FAD2 Desaturase Activity

Previous reports showed that some natural variations in FAD2 orthologs resulted in an elevated C18:1/C18:2 ratio in oil seed crops with C18:2 as the major fatty acid [10,11,14,23,24,25]. Thus it is feasible to find some natural FAD2 dysfunction variants in *Idesia ploycarpa*. A small population of five-year-old *Idesia ploycarpa* trees were examined. 32 Single nucleotide polymorphisms (SNPs) were found throughout the CDS region of IpFAD2s. The association between SNPs and the amino acid changes in IpFAD2s was summarized in Table 1. The SNPs caused synonymous mutations in the IpFAD2-1 CDS region, while the SNPs in IpFAD2-2 resulted in changes to five amino acids. The five IpFAD2-2 variants were named IpFAD2-2V1 (Y54S, V164I and V243M), IpFAD2-2V2 (Y54S), IpFAD2-2V3 (A69V), IpFAD2-2V4 (Q115/R and V164I), and IpFAD2-2V5 (V164I and V243M) (Supplementary Figure S4). Two of the five SNPs in IpFAD2-3 resulted in changes to two amino acids. The IpFAD2-3 variant was named as IpFAD2-3V1 (V253I and G376C) (Supplementary Figure S5). Seven of twelve SNPs in IpFAD2-4 resulted in changes to seven amino acids and the IpFAD2-4 variant was named a IpFAD2-4V1 (C151S, F164S, L172F, E289D and C365Y) and IpFAD2-4V2 (P31L, A71V, C151S and F164S) (Supplementary Figure S5).

Since SNPs caused the changes to amino acids in IpFAD2-2, IpFAD2-3, and IpFAD2-4, it is interesting to determine whether these amino acid variations affect desaturase activity of the three proteins. The constructs harboring wild type or FAD2 variants of IpFAD2-2, IpFAD2-3 and IpFAD2-4 were transformed into yeast *S. cerevisiae* INVSc1 and fatty acid composition was examined. The SNPs in IpFAD2-3s brought about the variations of two amino acids (V253I and G376C) in IpFAD2-3V1 (Table 1).

**Figure 5:**
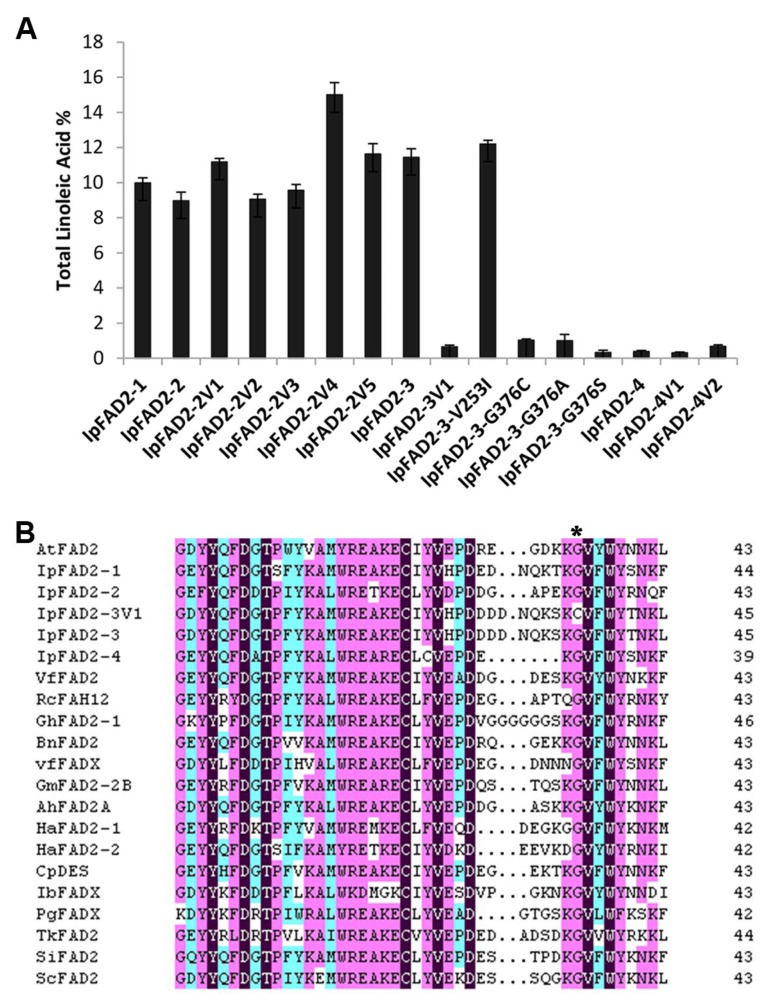
Total C18:2 accumulation in yeast transformed with different FAD2 alleles from *Idesia polycarpa*. (**A**) Total LA accumulation in transgenic yeast expressing natural FAD2 alleles and site-directed mutations from *Idesia polycarpa*. (**B**) Alignment of C-terminal amino acids of 21 FAD2 genes. The GenBank accession numbers of the proteins presented in this figure are shown in Figure 2. The asterisk indicates the conserved Glycine residue changed in IpFA2-3V1. The shading colors represent the identity level of amino acids. Black, magenta, and cyan indicate 100%, ~90%, and ~55% identity, respectively.

As shown in Figure 5A, the variations occurring in IpFAD2-2 and IpFAD2-4 have little effects on its desaturase activity as compared with their corresponding wild type form, whereas the two amino acid substitutions (V253I and G376C) severely affected the activity of IpFAD2-3V1 since the C18:2 content in IpFAD2-3V1 was only less than 10% of the wild type (Figure 5A). To ascertain which amino acid is responsible for this result, we performed single site mutation on IpFAD2-3 and obtained two *IpFAD2-3* mutants containing a single mutation with V253I or G376C. The G376C mutation caused the dramatic decreasing activity of IpFAD2-3, the percentage of C18:2 was greatly reduced to 8.3% of wild type (Figure 5A, Supplementary Figure S3). The activity of the variant containing V253I mutation was only slightly affected (Figure 5A, Supplementary Figure S3). These results suggested that Gly376 is essential for IpFAD2-3 activity. Here we noticed that Glycine was changed to Cysteine and thus deduced that the redox status might be concerned with the altered activity. To test this possibility, we replaced Gly376 with either alanine or serine. Our results showed that both replacements caused markedly decreased activity (Figure 5A). These results further illustrate the importance of G376 residues.

To find out the reasons why the substitution of the conserved glycine severely affects the activity of IpFAD2-3, we carefully examined plant FAD2 protein structure. This glycine residue is highly conserved across plant FAD2 proteins (Figure 5B). It is far away from the catalytic center consisting of three histidine-rich motifs [26], but is at -9 position relative to the C-terminus and adjacent to the mini ER retrieval sequence motif (Φ-X-X-K/R/D/E-Φ-COOH, YTNKL in the case of IpFAD2-3) [19]. Thus we hypothesized that the impacts of the glycine residue on IpFAD2-3 might act through disturbing the precise location of IpFAD2-3. To test this hypothesis, we made IpFAD2-3-GFP and IpFAD2-3-G376C-GFP constructs and co-transformed them with an ER membrane marker (CD3-959) into tobacco epidermal cells. Each fluorescent fusion protein was co-localized with the ER membrane marker CD3-959 (Supplementary Figure S6), consistent with the expression pattern of the wild type protein, indicating that G376C does not interrupt the localization of IpFAD2-3 and its impacts on enzyme activity could not act through mis-localizing the protein. The GFP fluorescent signals of tobacco epidermal cells containing either of IpFAD2-3-GFP or IpFAD2-3-G376C-GFP were similar to each other, suggesting that G376C does not affect IpFAD2-3 at protein level (Supplementary Figure S6).

### 2.4. Gly376 Has Different Effects on the Activity of FAD2 Proteins among Different Species

**Figure 6:**
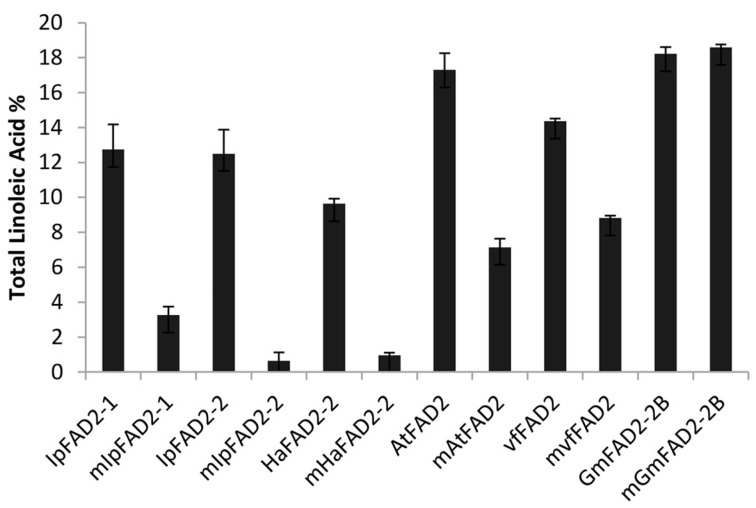
Total C18:2 accumulation in yeast containing FAD2 orthologs from different species. Levels of total C18:2 accumulation in yeast cells expressing plant FAD2s and the corresponding mutated form with the conserved glycine (G376, 375 or 374) substituted by cysteine. Ha, *Helianthus annuus*; At, *Arabidopsis thaliana*; Vf, *Vernicia fordii*; Gm, *Glycine max*. The corresponding mutated residues are: IpFAD2-1, G376C; IpFAD2-2, G374C; HaFAD2-2, G375C; AtFAD2, G375C; VfFAD2, G375C; GmFAD2-2B, G375C. “m” in front of each protein name represents the mutated form of the corresponding FAD2. Mean ± SD; *n* = 3.

Since the highly conserved Gly376 is important for IpFAD2-3 activity, we wondered if it has a conserved function in all IpFAD2 proteins and in FAD2 proteins from other species. Firstly, we introduced the mutation into IpFAD2-1 and IpFAD2-2. The mIpFAD2-1 and mIpFAD2-2 variants display markedly reduced desaturase activity as compared with wild type, suggesting that the conserved glycine is also required for enzyme activity of both IpFAD2-1 and IpFAD2-2 (Figure 6).

To check if the conserved glycine also has similar roles across plant FAD2 proteins, we cloned *FAD2* genes from *Helianthus annuus*, *Arabidopsis thaliana*, *Vernicia fordii* and *Glycine max,* which are *HaFAD2-2, AtFAD2, VfFAD2* and *GmFAD2-2B* accordingly, and also replaced this glycine with cysteine in these FAD2 proteins. The wild type and mutated constructs were then transformed into yeast and FA composition was detected by GC. As shown in Figure 6, the yeast cells containing each wild-type *FAD2s* produced a certain amount of C18:2, ranging from 9% to 18% of total fatty acids, indicating that all these selected FAD2s have functional Δ^12^ oleate desaturase activity in heterologous yeast cells. We then checked the C18:2 levels of the yeast cells containing the mutated constructs and found that the effects of the mutation (G376/C) on the activity of FAD2 proteins varied among different proteins. The levels of C18:2 in yeast cells containing the mutated HaFAD2-2 were markedly decreased to less than 10% of wild type (Figure 6). The activity of mutated AtFAD2 and VfFAD2 only showed moderate reduction since the mutants showed about 58% and 38% reductions. The mutated glycine in GmFAD2-2B has no effects on the production of C18:2 (Figure 6). Taken together, these results suggested that the effects of the highly conserved glycine at 376 position of IpFAD2-3 on plant FAD2 activity probably act in a species/protein-specific manner.

## 3. Discussion

The *FAD2* gene was first identified in Arabidopsis [27], since then many *FAD2* genes have been cloned from different plants [26,28,29,30,31,32,33]. To date, none of the microsomal Δ^12^ fatty acid desaturases associated with C18:2 have been identified in *Idesia ploycarpa*. Here we isolated four *IpFAD2* genes. Deduced amino acid sequences alignment showed that the four IpFAD2s shared 69.5–78.5% identity with AtFAD2, The *Idesia polycarpa* fruit, consisting of pericarp and seed, produces large amounts of fatty acids, among which about 83.92% of C18:2 is present in pericarp oils, that far exceeds seed oils [5]. To gain insight into the oil accumulation mechanism in pericarp and seed, we studied the expression patterns of four *IpFAD2* paralogues in these two organs by real time-PCR. Each gene shows distinct expression patterns (Figure 3). *IpFAD2-2* is highly expressed in both organs, its transcripts far exceed those of other *IpFAD2* genes, suggesting that *IpFAD2-2* might be a major gene responsible for C18:2 production in the fruit of *Idesia polycarpa*. The expression levels of *IpFAD2-2* varied between two parts, i.e., higher in pericarps and lower in seeds, suggesting its different roles in the two organs. Taken together, *IpFAD2-2* might be a candidate for genetically modifying the ratio of C18:1/C18:2 in *Idesia ploycarpa* fruits in the future. As compared with *IpFAD2-2*, the expression levels of the other three *IpFAD2* genes are very low in fruit (Figure 3). They also exhibit the distinct expression patterns found in pericarps and seeds. The expression of *IpFAD2-3* displays little differences between the two organs while *IpFAD2-1* was preferably expressed in the pericarp. The different expression patterns of these *IpFAD2* genes might cause the different ratios of C18:1/C18:2 in pericarps and seeds.

To identify the activity of IpFAD2s, we cloned these four genes and introduced them into yeast cells. With the exception of IpFAD2-4, the three other IpFAD2 proteins efficiently converted C18:1 to C18:2 in the yeast system (Figure 4). To investigate the reason why IpFAD2-4 displayed low activity, we carefully compared the amino acid sequences of IpFAD2-4 with that of the other proteins and found that IpFAD2-4 shared 85.1% with *IpFAD2-2* (Figure 1 and Figure 2), suggesting that both genes might be derived from duplication events. RT-PCR results showed that it displayed similar patterns to *IpFAD2* though its expression levels are far lower than the latter. But the two proteins showed distinct activity in yeast cells. To precisely compare the activity of these proteins, we need to transform it into plants for functional identification in the near future.

With the rapid advances in biotechnology, genetic engineering has been widely used in identifying gene function or modifying plant quality in the lab due to its simplicity and easy-of-use. But its extensive application in nature is largely restricted since the impacts of genetically modified plants on nature are unpredictable. Natural variants have endured long-term natural selection and natural mutation is thus more reliable, stable, less toxic, and desirable for variety breeding. Natural variations in FAD2 coding region correlated with the C18:1 content have been identified from different plants. D150N and H101D from peanut, S117N and P137R from soybean have been shown to decrease the activity of FAD2 [10,34]. Most substitutions were in or near the His-box, which makes up the catalytic center. Here we checked 32 SNP sites present in FAD2 coding region among 30 *Idesia ploycarpa* natural variants. These SNP sites caused fourteen amino acid substitutions and twelve IpFAD2 alleles. Our data revealed that only the substitution of the highly conserved Gly376 severely affects the IpFAD2-3 activity (Figure 5A), suggesting its important role for IpFAD2-3. Its replacement also severely disrupted the activity of IpFAD2-1 and IpFAD2-2 (Figure 5A). We also expand its mutation to other plant FAD2 proteins and found that this glycine more or less affects the function of most of the tested plant FAD2 proteins except for GmFAD2-2B (Figure 6). Till now it is unknown how the glycine mutation causes the reduced products catalyzed by IpFAD2-3 proteins. Its mutation does not to affect the protein levels since the mutated protein can be normally expressed as shown in Supplementary Figure S6. The effects of this conserved glycine on enzyme activity might be associated with other unknown factors such as protein structure formation, phosphorylation and etc. All in all, the identified G376 in IpFAD2-3 could be a potential site for the manipulation of the desaturase activity of IpFAD2 by genome editing in the near future, and it will be applicable for genetically improving crop quality.

## 4. Materials and Methods

### 4.1. Plant Materials

The fresh leaves of 26 five-year-old *Idesia ploycarpa* female trees, which are growing at Huanggang, Hubei province, China, were collected, mixed and quickly frozen in liquid nitrogen for further RNA extraction. Col-0 *Arabidopsis thaliana* are growing in greenhouse condition. Sunflower (*Helianthus annuus*), tung tree (*Vernicia fordi*), and soybean (*Glycine max*) were collected from Wuhan Botanical Garden, Wuhan, China. The 80 days after pollination (DAP) fruits of *Idesia ploycarpa* cultivar 76A were quickly frozen in liquid nitrogen and stored at −80 °C until use.

### 4.2. Total RNA Extraction and Complementary DNA Synthesis

Total RNA was isolated from 100 mg of frozen leaves and seeds and pericarps of 80 DAP *Idesia ploycarpa* fruit with Trizol reagent (Life Technologies Corporation, Carlsbad, CA, USA) according to the manufacturer’s protocol. RNA concentration was determined by NanoDropTM spectrophotometer ND2000 (Thermo Fisher Scientific, Wilmington, DE, USA). Total RNA was then treated with DNase I (Thermo Fisher Scientific, Wilmington, DE, USA) to eliminate residue DNA. About 500 ng DNA-free RNA was used as a template for first-strand complementary DNA (cDNA) synthesis. Reverse-transcription was performed with the M-MLV Reverse Transcriptase (Promega, Madison, WI, USA) and oligo(dT)_20_ primer (Tsingke, Wuhan, China).

### 4.3. Gene Cloning and Sequence Analysis

Using leaf cDNA as template, we cloned the coding sequences of different *IpFAD2* with corresponding primers listed in Supplementary Table S2. The primers were designed according to the sequence submitted by Li et al. [5]. Due to lack of upstream sequence information, a degenerate primer was designed as the forward primer according to sequence homology used for *IpFAD2-4* cloning. The FAD2 fragments obtained were cloned into the pESC-his vector (Alilent Technologies, Santa Clara, CA, USA) directly and then sequenced. Multiple sequence alignments were performed using DNAman software. For phylogenetic relationship analysis, the protein sequences of IpFAD2 protein sequence and a number of plant FAD2 homologs were aligned with MAFFT v7.154b [35]. Maximum-likelihood (ML) tree was generated using FastTree v2.1.7 [36] and was visualized using FigTree (Available online: http://tree.bio.ed.ac.uk/software/figtree/).

### 4.4. Real-Time Quantitative PCR

Gene expression analysis was performed by RT-PCR using Applied Biosystems 7500 Fast Real-Time PCR System (Thermo Fisher Scientific, Wilmington, DE, USA). Primers with Tm (melting temperature) 60 °C and 18–20 bp in length were designed by Primer 3 (Supplementary Table S2). IpEF1A was selected as the internal reference gene. PCR reaction mix (20 μl per well) contained 10 μl TB Green Premix Ex Taq II (TliRNaseH Plus) (2X) (Takara, Tokyo, Japan), 0.8 μl forward and reverse primers (10 μM) (Tsingke, Wuhan, China), 0.4 μl ROX Reference Dye II (Takara, Tokyo, Japan), 50 ng cDNA and RNA-free water. The two-step thermal cycling conditions were 95 °C for 30 s, followed by 40 cycles of 95 °C for 5 s, 60 °C for 34 s. Corresponding gene expression level was analyzed with the 2-ΔΔ*C*t method. Elongation factor 1-alpha was used as the internal control to normalize the relative amount of mRNAs for all samples.

### 4.5. Site-Directed Mutagenesis

Mutagenesis was done according to the fast mutagenesis system (Transgen, Beijing, China). In brief, the mutated plasmids were amplified with two primers containing the mutations (Supplementary Table S2) using the TransStart FastPfu DNA polymerase (Transgen, Beijing, China). The PCR conditions were as follows: initial denaturation at 94 °C for 5 min, followed by 25 cycles of 94 °C for 20 s, 55 °C for 20 s, and 72 °C for 3 min, final extension at 72 °C for 10 min. The amplicons were subsequently digested with DMT (Transgen, Beijing, China) enzyme for eliminating the methylated parental plasmid and then purified from agrose gels. The purified products were transformed into DMT competent cells (Transgen, Beijing, China). The mutated clones selected on plates containing antibiotics were verified by sequencing.

### 4.6. Yeast Transformation and Heterologous Expression of IpFAD2 Variants

Constructs containing the *IpFAD2* genes were transformed into *Saccharomyces cerevisiae* INVSc1 (Invitrogen, Carlsbad, CA, USA) by the LiAc/SS carrier DNA/PEG method. Transformants were incubated in yeast nitrogen base (YNB) liquid medium at 28 °C for 36 h with rotary shaking at 180 rpm and then spread on synthetic defined medium without histidine (SD-his, Clonetech, Mountain View, CA, USA) solidified medium supplemented with glucose. The colonies growing on SD-his medium were then cultured in SD-his liquid medium for another two days and then centrifuged. The pellets were washed with distilled water twice and sub-cultured in SD-his liquid medium containing galactose (2%, *w*/*v*) for 48 h. Yeast cells were collected for fatty acid analysis.

### 4.7. Analysis of Fatty Acid Composition in Yeast

The induced yeast cells transformed with *FAD2* cDNA fragments were pelleted and washed with distilled water twice, and total lipids were extracted with hexane and methylated with 5M KOH-methanol. The heptadecanoic acid methyl ester (C17:0) was used as the internal standard. Fatty acid methyl esters were measured by gas chromatography with an Agilent 7820A (Alilent Technologies, Santa Clara, CA, USA). The samples were separated on an Agilent DB-23 capillary column (Alilent Technologies, Santa Clara, CA, USA). The column temperature was programmed with an initial temperature of 180 °C for 1 min, ramping at 3 °C/min to 240 °C, and then holding for 39 min.

### 4.8. Subcellular Localization Assay

The coding sequences of IpFAD2-3 and IpFAD2-3V1 were amplified with specific primers (Supplemental Table S2). The amplified fragments were cloned into the PMDC83 vector, which generated Pro35S::IpFAD2-3::GFP and Pro35S::IpFAD2-3V1::GFP fusion constructs. The obtained plasmids were transferred into *Agrobacterium tumefaciens* (GV3101) using the freeze-thaw method, and subsequently transformed into leaves of *Nicotiana benthamiana* by infiltration. To precisely localize which compartments the FAD2 proteins reside in, CD3-959 (35S-ER-mCherry), an ER marker [37] was co-transformed with the FAD proteins. The fluorescent signals generated by GFP and mCherry fusion proteins were observed by confocal microscopy.

## 5. Conclusions

We identified four *FAD2* homologs from fruits of *Idesia polycarpa*. Heterologous expression in yeast showed that three IpFAD2s have strong Δ^12^ fatty acid desaturase activity. Natural variation together with site-directed mutagenesis analysis reveals one natural variation (G376C in IpFAD2-3) that strongly hinders the catalytic activity of IpFAD2-3. Even though this amino acid is highly conserved among plant FAD2 proteins, the effects of its mutation on the Δ^12^ oleate desaturase activity of tested FAD2 proteins are different. Our findings will be helpful to advance understanding the roles of FAD2 proteins in woody plants and also provide a new potential site of IpFAD2s for modifying the ratio of C18:1/C18:2 of *Idesia polycarpa* fruit in the future through genetic engineering. Further characterization of the mechanisms of the effects of G376C substitutions in different FAD2s, either at the enzyme activity level or on other modulatory molecules, is currently under way in our group.

## Figures and Tables

**Table 1:** Single nucleotide polymorphisms (SNPs) in the coding region of fatty acid desaturase 2 (*FAD2*) genes from *Idesia ploycarpa*.

*FAD2* Gene	SNPSite	Amino Acid Position	SNP Mutation	Amino Acid Mutation	Mutation Type
*IpFAD2-1*	201	67	TAT→TAC	Tyr	S
(1158 bp)	729	243	GCA→GCG	Ala	S
	765	255	TAT→TAC	Tyr	S
*IpFAD2-2*	161	54	TAT→TCT	Tyr→Ser	N
(1149 bp)	206	69	GCC→GTC	Ala→Val	N
	344	115	CAG→CGG	Gln→Arg	N
	372	124	ATC→ATT	Ile	S
	399	133	TAC→TAT	Tyr	S
	490	164	AGT→AAT	Ser→Asn	N
	612	204	CGA→CGC	Arg	S
	624	208	CAC→CAT	His	S
	727	243	GTG→ATG	Val→Met	N
	1041	347	GAC→GAT	Asp	S
	1092	364	GTT→GTG	Val	S
	1113	371	CCA→CCC	Pro	S
*IpFAD2-3*	690	230	GGC→GGT	Gly	S
(1158 bp)	696	232	CTC→CTT	Leu	S
	729	243	GTC→GTA	Val	S
	757	253	GTT→ATT	Val→Ile	N
	1126	376	GGC→TGC	Gly→Cys	N
*IpFAD2-4*	92	31	CCC→CTC	Pro→Leu	N
(1143 bp)	212	71	GCC→GTC	Ala→Val	N
	279	93	CTA→CTC	Leu	S
	451	151	TGC→AGC	Cys→Ser	N
	480	160	CCA→CCG	Pro	S
	491	164	TTC→TCC	Phe→Ser	N
	514	172	CTC→TTC	Leu→Phe	N
	531	177	CCT→CCA	Pro	S
	867	289	GAA→GAT	Glu→Asp	N
	999	333	GCA→GCT	Ala	S
	1002	334	ACT→ACA	Thr	S
	1094	365	TGT→TAT	Cys→Tyr	N

S represents synonymous, N represents nonsynonymous.

## Supplementary Materials

Supplementary materials can be found at http://www.mdpi.com/1422-0067/19/12/3932/s1.
