# Supplementary material for: A Conserved Glycine Is Identified to be Essential for Desaturase Activity of IpFAD2s by Analyzing Natural Variants from Idesia polycarpa
Source: Int J Mol Sci. 2018 Dec 7;19(12):3932. doi: 10.3390/ijms19123932 (PMC6321622; doi:10.3390/ijms19123932)
Supplement: Supplementary file 1 [file ijms-19-03932-s001.pdf]

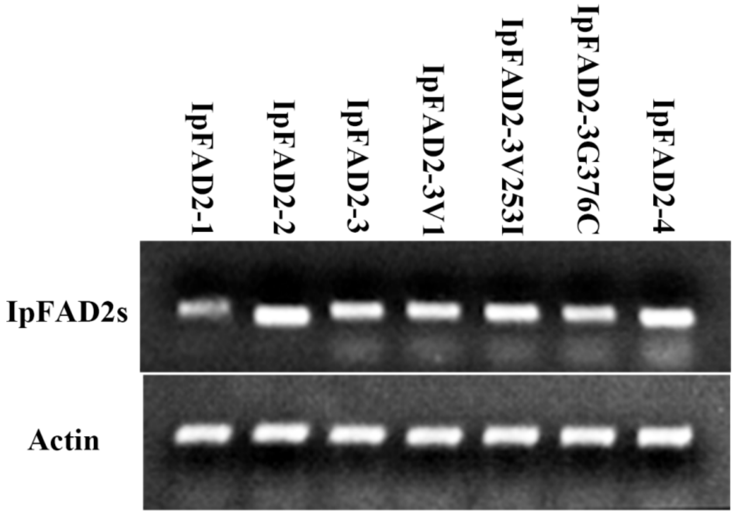

Supplementary Figure S1. *IpFAD2s* transcripts in transgenic yeast lines.

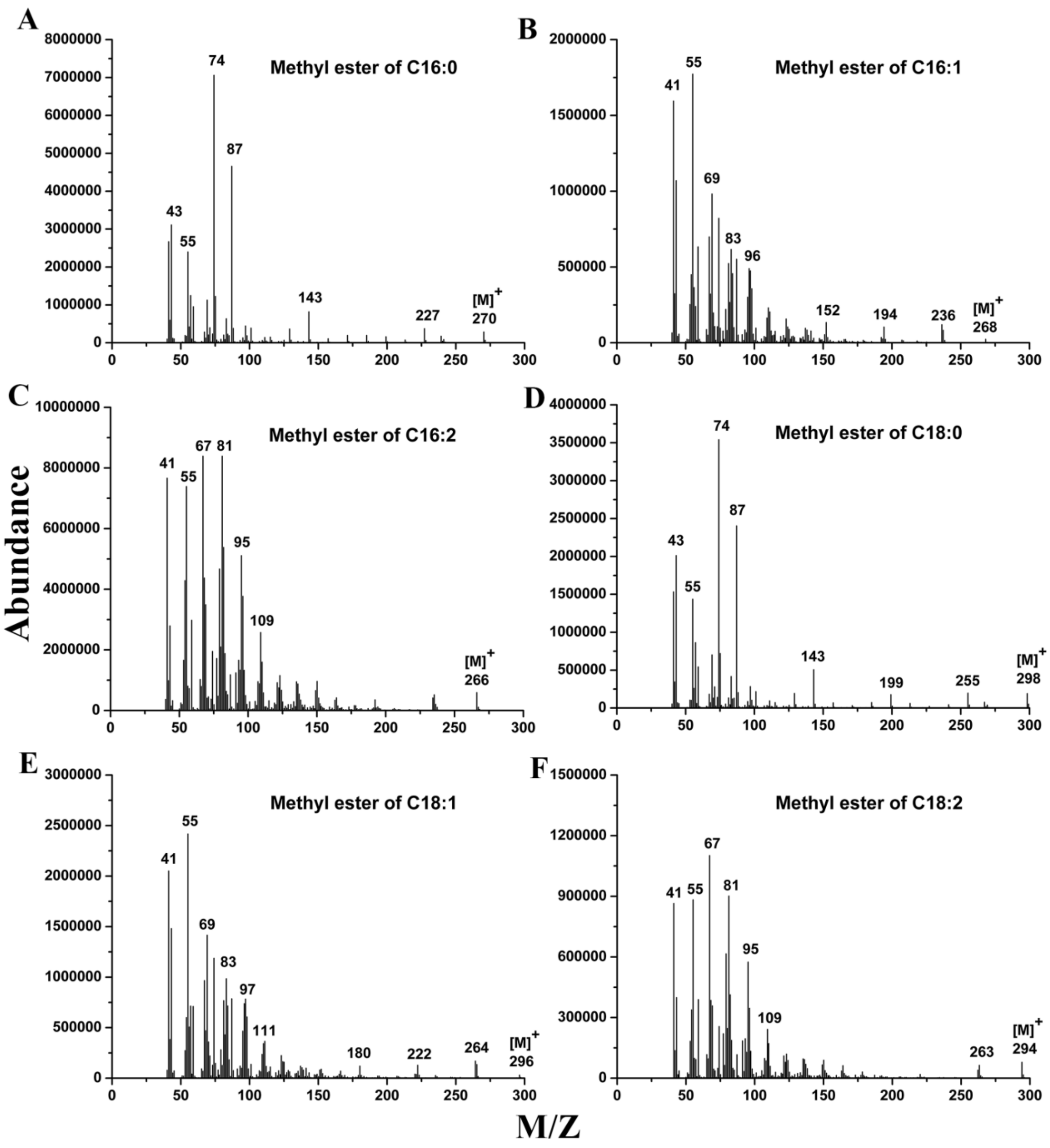

Supplementary Figure S2. Mass spectrum of fatty acid methyl esters from yeast cells transformed with vector harboring *IpFAD2* genes. Diagnostic fragment ions are labeled in boldface. $[M]^+$, Molecular ion.

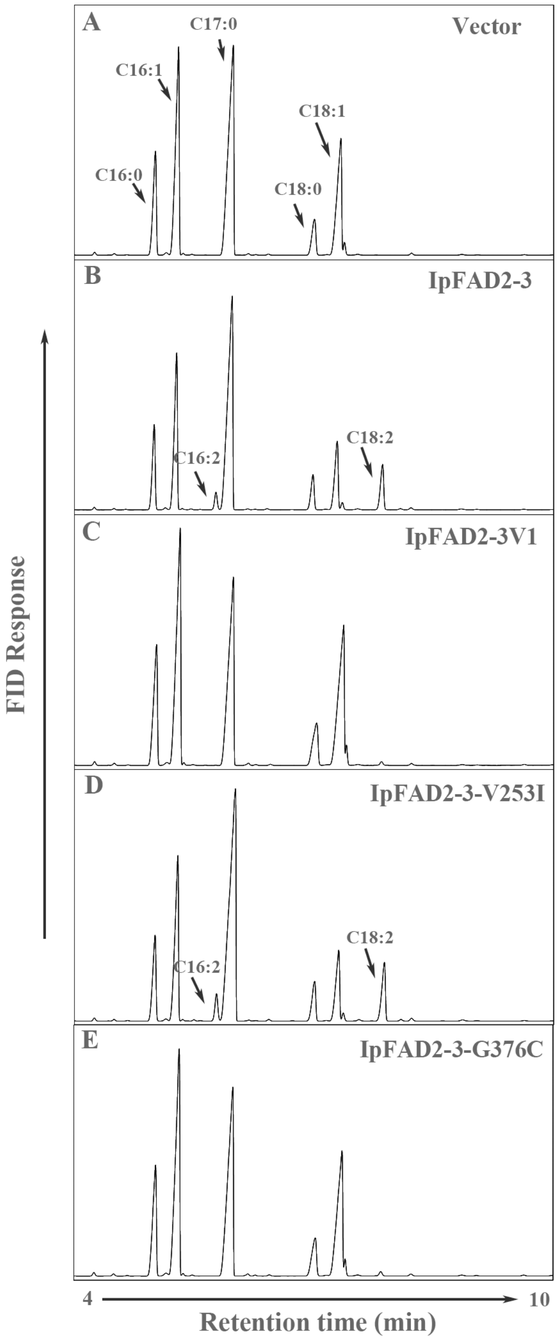

Supplementary Figure S3. GC analysis of FAMEs isolated from yeast cells expressing IpFAD2-3 and its mutant forms. The FAMEs of total lipid were extracted from yeast transformed with empty vector control pESC-his (A), IpFAD2-3 (B), IpFAD2-3V1 (C), IpFAD2-3-V253I (D) and IpFAD2-3-G376C under induction conditions and then analyzed by GC/FID. The peaks of major fatty acid are indicated by arrows. The heptadecanoic acid methyl ester (C17:0) is used as the internal standard.

```
IpFAD2-2     MGANGVFASSAKHEGKESRIKRMPYAKPPFTLGKIKKAIPPHCFERSLLRSFSYVGYDLC    60
IpFAD2-2V1   MGANGVFASSAKHEGKESRIKRMPYAKPPFTLGKIKKAIPPHCFERSLLRSFSSVGYDLC    60
IpFAD2-2V2   MGANGVFASSAKHEGKESRIKRMPYAKPPFTLGKIKKAIPPHCFERSLLRSFSSVGYDLC    60
IpFAD2-2V3   MGANGVFASSAKHEGKESRIKRMPYAKPPFTLGKIKKAIPPHCFERSLLRSFSYVGYDLC    60
IpFAD2-2V4   MGANGVFASSAKHEGKESRIKRMPYAKPPFTLGKIKKAIPPHCFERSLLRSFSYVGYDLC    60
IpFAD2-2V5   MGANGVFASSAKHEGKESRIKRMPYAKPPFTLGKIKKAIPPHCFERSLLRSFSYVGYDLC    60
                                                                  *

IpFAD2-2     ISFLLCYIAITYFHLLPSPLAYIAWPIYWILQGCILTGVWVIAHECGHHAFSDYQWVDDT    120
IpFAD2-2V1   ISFLLCYIAITYFHLLPSPLAYIAWPIYWILQGCILTGVWVIAHECGHHAFSDYQWVDDT    120
IpFAD2-2V2   ISFLLCYIAITYFHLLPSPLAYIAWPIYWILQGCILTGVWVIAHECGHHAFSDYQWVDDT    120
IpFAD2-2V3   ISFLLCYIVITYFHLLPSPLAYIAWPIYWILQGCILTGVWVIAHECGHHAFSDYQWVDDT    120
IpFAD2-2V4   ISFLLCYIAITYFHLLPSPLAYIAWPIYWILQGCILTGVWVIAHECGHHAFSDYRWVDDT    120
IpFAD2-2V5   ISFLLCYIAITYFHLLPSPLAYIAWPIYWILQGCILTGVWVIAHECGHHAFSDYQWVDDT    120
                     *                                             *

IpFAD2-2     VGLILHSALFVPYFSWKYSHRRHHSNTGSLERDEVFVPKPKSRVAWYSKYLNNPPGRALS    180
IpFAD2-2V1   VGLILHSALFVPYFSWKYSHRRHHSNTGSLERDEVFVPKPKSRIAWYSKYLNNPPGRALS    180
IpFAD2-2V2   VGLILHSALFVPYFSWKYSHRRHHSNTGSLERDEVFVPKPKSRVAWYSKYLNNPPGRALS    180
IpFAD2-2V3   VGLILHSALFVPYFSWKYSHRRHHSNTGSLERDEVFVPKPKSRVAWYSKYLNNPPGRALS    180
IpFAD2-2V4   VGLILHSALFVPYFSWKYSHRRHHSNTGSLERDEVFVPKPKSRVAWYSKYLNNPPGRALS    180
IpFAD2-2V5   VGLILHSALFVPYFSWKYSHRRHHSNTGSLERDEVFVPKPKSRIAWYSKYLNNPPGRALS    180
                                                        *

IpFAD2-2     LAVTLLLGWPLYLAFNVSGRPYDRFACHYDPHGPIYSDRERLQIYISDLGIFAATFVLYN    240
IpFAD2-2V1   LAVTLLLGWPLYLAFNVSGRPYDRFACHYDPHGPIYSDRERLQIYISDLGIFAATFVLYN    240
IpFAD2-2V2   LAVTLLLGWPLYLAFNVSGRPYDRFACHYDPHGPIYSDRERLQIYISDLGIFAATFVLYN    240
IpFAD2-2V3   LAVTLLLGWPLYLAFNVSGRPYDRFACHYDPHGPIYSDRERLQIYISDLGIFAATFVLYN    240
IpFAD2-2V4   LAVTLLLGWPLYLAFNVSGRPYDRFACHYDPHGPIYSDRERLQIYISDLGIFAATFVLYN    240
IpFAD2-2V5   LAVTLLLGWPLYLAFNVSGRPYDRFACHYDPHGPIYSDRERLQIYISDLGIFAATFVLYN    240

IpFAD2-2     IAVSQGLAFLICIYGVPLLIVNGFLVTITYLQHTHPSLPHYDSSEWEWLRGALVTMDRDY    300
IpFAD2-2V1   IAMSQGLAFLICIYGVPLLIVNGFLVTITYLQHTHPSLPHYDSSEWEWLRGALVTMDRDY    300
IpFAD2-2V2   IAVSQGLAFLICIYGVPLLIVNGFLVTITYLQHTHPSLPHYDSSEWEWLRGALVTMDRDY    300
IpFAD2-2V3   IAVSQGLAFLICIYGVPLLIVNGFLVTITYLQHTHPSLPHYDSSEWEWLRGALVTMDRDY    300
IpFAD2-2V4   IAVSQGLAFLICIYGVPLLIVNGFLVTITYLQHTHPSLPHYDSSEWEWLRGALVTMDRDY    300
IpFAD2-2V5   IAMSQGLAFLICIYGVPLLIVNGFLVTITYLQHTHPSLPHYDSSEWEWLRGALVTMDRDY    300
               *

IpFAD2-2     GILNKVFHNITDTHVTHHLFSTMPHYHATEATKAIKPILGEFYQFDDTPIYKALWRETKE    360
IpFAD2-2V1   GILNKVFHNITDTHVTHHLFSTMPHYHATEATKAIKPILGEFYQFDDTPIYKALWRETKE    360
IpFAD2-2V2   GILNKVFHNITDTHVTHHLFSTMPHYHATEATKAIKPILGEFYQFDDTPIYKALWRETKE    360
IpFAD2-2V3   GILNKVFHNITDTHVTHHLFSTMPHYHATEATKAIKPILGEFYQFDDTPIYKALWRETKE    360
IpFAD2-2V4   GILNKVFHNITDTHVTHHLFSTMPHYHATEATKAIKPILGEFYQFDDTPIYKALWRETKE    360
IpFAD2-2V5   GILNKVFHNITDTHVTHHLFSTMPHYHATEATKAIKPILGEFYQFDDTPIYKALWRETKE    360

IpFAD2-2     CLYVDPDDGAPEKGVFWYRNQF                                          382
IpFAD2-2V1   CLYVDPDDGAPEKGVFWYRNQF                                          382
IpFAD2-2V2   CLYVDPDDGAPEKGVFWYRNQF                                          382
IpFAD2-2V3   CLYVDPDDGAPEKGVFWYRNQF                                          382
IpFAD2-2V4   CLYVDPDDGAPEKGVFWYRNQF                                          382
IpFAD2-2V5   CLYVDPDDGAPEKGVFWYRNQF                                          382
```

Supplementary Figure S4. Multiple alignments of predicted amino acid sequences of IpFAD2-2 variants.

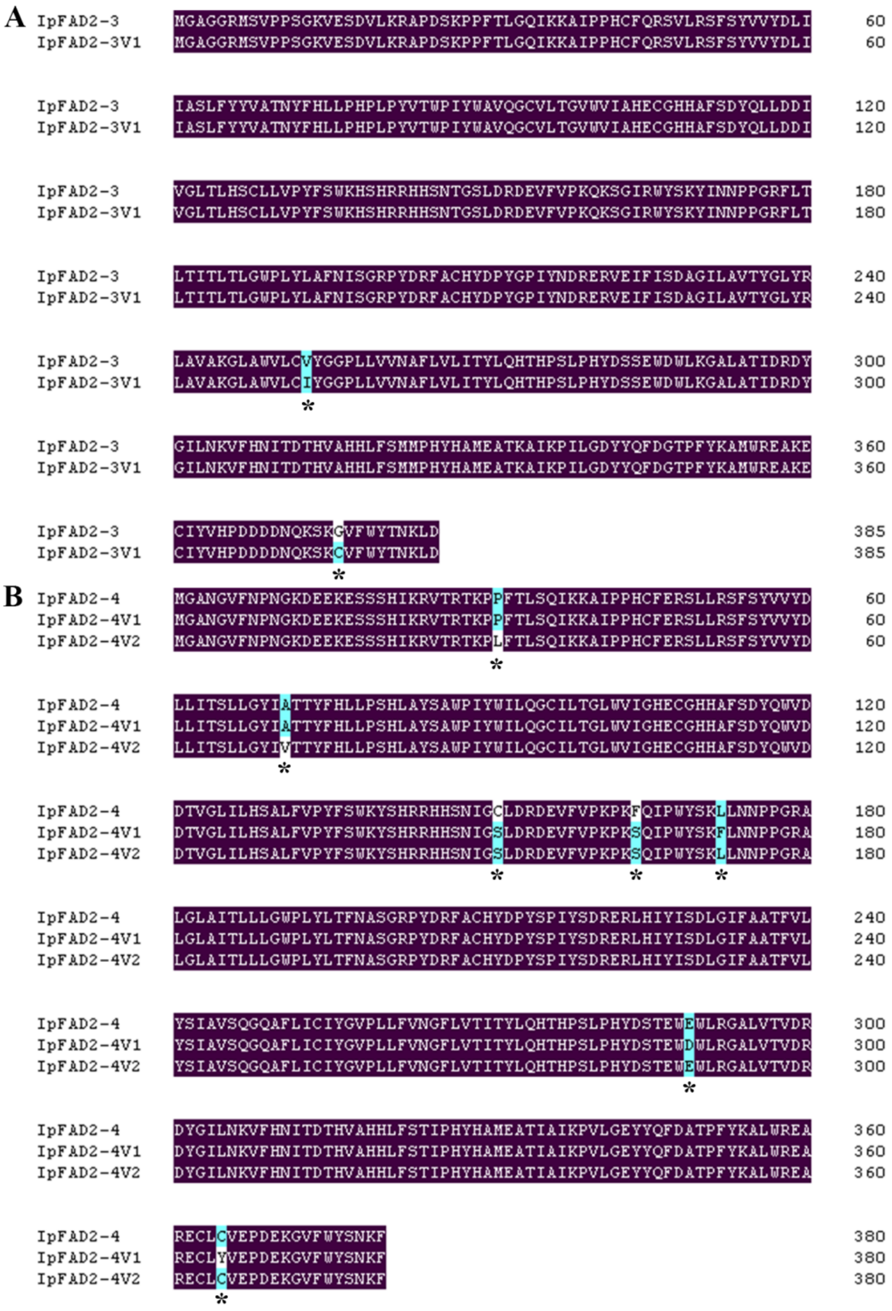

Supplementary Figure S5. Alignment of predicted amino acid sequences of IpFAD2-3 variants (A) and IpFAD2-4 variants (B).

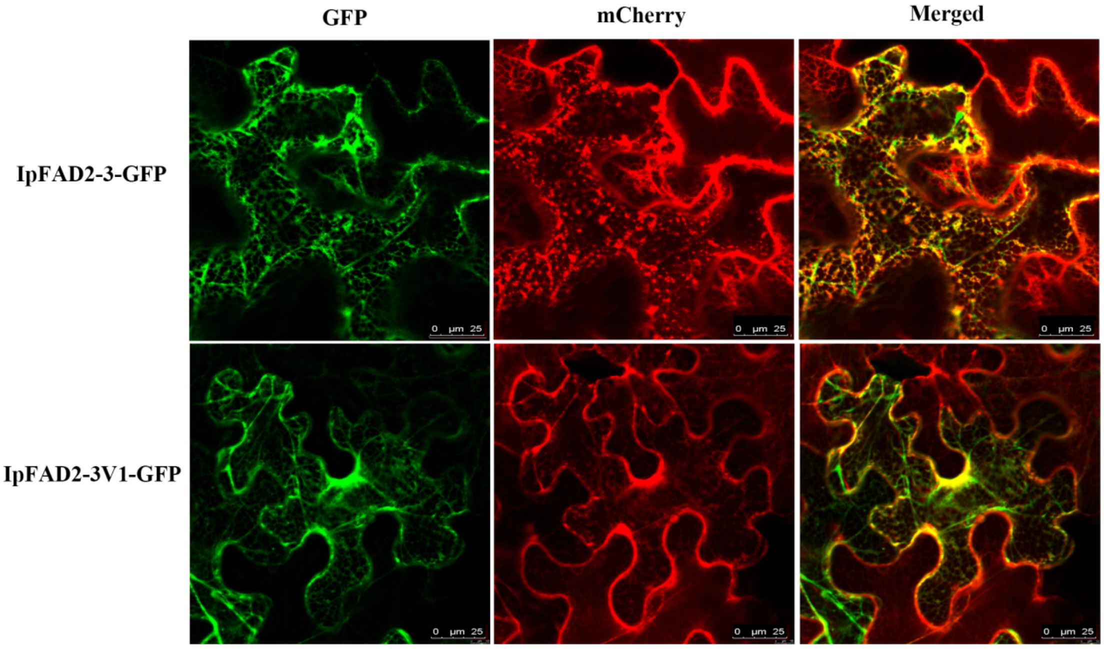

Supplementary Figure S6. Subcellular localization of IpFAD2-3 and IpFAD2-3V1. Subcellular localization of IpFAD2-3 and IpFAD2-3V1 with GFP fusion protein in *Nicotiana benthamiana* leaves. Images were taken with a confocal laser scanning microscope. Green, GFP fluorescence image; mCherry, red fluorescent image; Merged, the merged signals simultaneously excited by GFP and mCherry fusion proteins. Bars: 25 µm.

Supplementary Table S1. Sequence identity of the deduced amino acids in FAD2 genes.

	IpFAD2-2	IpFAD2-3	IpFAD2-4	AtFAD2	GmFAD2-2B	HaFAD2-2	VfFAD2
IpFAD2-1	73.90%	93.02%	73.13%	76.74%	83.46%	73.90%	85.53%
IpFAD2-2		73.13%	82.95%	70.03%	74.42%	69.77%	74.16%
IpFAD2-3			71.83%	76.49%	82.69%	74.16%	85.79%
IpFAD2-4				67.70%	72.87%	66.15%	72.09%
AtFAD2					76.49%	71.83%	77.52%
GmFAD2-2B						73.39%	82.95%
HaFAD2-2							73.90%

The GenBank accession numbers of the proteins represented in this figure are shown in Figure 2.

Supplementary Table S2. Primers used in this study.

IpFAD2-1-F	CGGAATTCCATGGGTGCTGGTGGAAGAAT
IpFAD2-1-R	CGGACTAGTTCAATCAAATTTGTTCGAGTACC
IpFAD2-2-F	CGGAATTCCATGGGAGCCAATGGAGTGTTT
IpFAD2-2-R	CGGACTAGTTCAAAACTGGTTCCGGTACCAGAAC
IpFAD2-3-F	CGGAATTCCATGGGTGCTGGCGGTAGAATGT
IpFAD2-3-R	CGGACTAGTTTAATCCAATTTATTTGTGTACC
IpFAD2-4-F1	RWMRSKKMDYRRAACAATGGG
IpFAD2-4-F2	AATTTTTGAAAATTCGAATTCATGGGAGCCAATGGAGTGT
IpFAD2-4-R	TTGTAATCCATCGATACTAGTTTAAAACTTGTTACTGTACCAG AAAACCCCCT
AtFAD2-F	CGGAATTCCATGGGTGCAGGTGGAAGAAT
AtFAD2-R	CGGACTAGTTCATAACTTATTGTTGTACCAGT
VfFAD2-F	CGGAATTCCATGGCTTCCACCACTGCTCTT
VfFAD2-R	CGGACTAGTTCAAAACTTCTTGTTAT
GmFAD2-2B-F	CGGAATTCCATGGGGGCTGGTGGCCGAACTGCT
GmFAD2-2B-R	CGGACTAGTTCACAACTTATTGTTGTACCAAAAT
HaFAD2-2-F	CGGAATTCCATGGGTGCCGGCGGGCGGATGTCG
HaFAD2-2-R	CGGACTAGTTCATATCTTGTTACGGTACCAGTAAAC
mIpFAD2-1-F	ATAACCAGAAGACAAAAtGCGTATTTTGGT
mIpFAD2-1-R	aTTTTGTCTTCTGGTTATCTTCATCTGGAT
mIpFAD2-2-F	ATGACGGAGCTCCCGAAAAAtGCGTGTTCTGGT
mIpFAD2-2-R	aTTTTTCGGGAGCTCCGTCATCTGGATCCACAT
mAtFAD2-F	AGGGAAGGTGACAAGAAATGTGTGTACTGGT
mAtFAD2-R	ATTTCTTGTCACCTTCCCTGTCCGGTTCT
mVfFAD2-F	ATGGTGATGAAAGCAAATGTGTGTACTG
mVfFAD2-R	ATTTGCTTTCATCACCATCATCTGCCT
mGmFAD2-3-F	AGTACTCAGAGCAAATGTGTATTTTGGT
mGmFAD2-3-R	ATTTGCTCTGAGTACTTTGATCTGGCT
mHaFAD2-2-F	ATGAGGAGGTTAAGGATtGTGTTTACTGGT
mHaFAD2-2-R	aATCCTTAACCTCCTCATCTTTATCAACAT
IpFAD2-3-V253I-F	CGCTTGGGTTCTTTGTGTTTATGGAGGACCAT
IpFAD2-3-V253I-R	ATGGTCCTCCATAAACACAAAGAACCCAAGCG
IpFAD2-3-G376C-F	AACCAGAAGAGCAAAGGCGTATTTTGGT
IpFAD2-3-G376C-R	CTTTGCTCTTCTGGTTATCATCATCAT
IpFAD2-3-GFP-F	CGGACTAGTATGGGTGCTGGCGGTAGAAT
IpFAD2-3-GFP-R	AGGCGCGCCTATCCAATTTATTTGTGTACC
IpFAD2-1-qPCR-F	AAAGCCATTCCACCTCATTG
IpFAD2-1-qPCR-R	ATAACCCAAACGCCAGTCAG

IpFAD2-2-qPCR-F	CGGAGGCCACTAAAGCAATC
IpFAD2-2-qPCR-R	GCATCAAAACTGGTTCCGGT
IpFAD2-3-qPCR-F	CTTGGCCAGATCAAGAAAGC
IpFAD2-3-qPCR-R	AACTCCGGTGAGGACACATC
IpFAD2-4-qPCR-F	GTGGGTCGATGACACAGTTG
IpFAD2-4-qPCR-R	ATGGGATTTGGGACTTAGGC
IpEF1A-F	GCCTGGTATGGTTGTGACCT
IpEF1A-R	GGATCATCCTTAGAGTTAGA
G376S-F	ATAACCAGAAGAGCAAAAGCGTATTTTGGT
G376S-R	TTTTGCTCTTCTGGTTATCATCATCATCT
G376A-F	ATAACCAGAAGAGCAAAGCCGTATTTTGGT
G376A-R	GCTTTGCTCTTCTGGTTATCATCATCATCT
Actin-F	AGTTGCCCCAGAAGAACACC
Actin-R	TACCGGCAGATTCCAAACCC
